# Causal relationships between lung cancer and sepsis: a genetic correlation and multivariate mendelian randomization analysis

**DOI:** 10.3389/fgene.2024.1381303

**Published:** 2024-06-28

**Authors:** Jiejun Zhou, Youqian Zhang, Tian Yang, Kun Zhang, Anqi Li, Meng Li, Xiaojing Peng, Mingwei Chen

**Affiliations:** ^1^ Department of Respiratory and Critical Care Medicine, The First Affiliated Hospital of Xi’an Jiaotong University, Xi’an, China; ^2^ Health Science Center, Yangtze University, Jingzhou, Hubei, China

**Keywords:** causal association, genome-wide association study, lung cancer, mendelian randomization, sepsis

## Abstract

**Background:**

Former research has emphasized a correlation between lung cancer (LC) and sepsis, but the causative link remains unclear.

**Method:**

This study used univariate Mendelian Randomization (MR) to explore the causal relationship between LC, its subtypes, and sepsis. Linkage Disequilibrium Score (LDSC) regression was used to calculate genetic correlations. Multivariate MR was applied to investigate the role of seven confounding factors. The primary method utilized was inverse-variance-weighted (IVW), supplemented by sensitivity analyses to assess directionality, heterogeneity, and result robustness.

**Results:**

LDSC analysis revealed a significant genetic correlation between LC and sepsis (genetic correlation = 0.325, *p* = 0.014). Following false discovery rate (FDR) correction, strong evidence suggested that genetically predicted LC (OR = 1.172, 95% CI 1.083–1.269, *p* = 8.29 × 10^−5^, *P*_*fdr*_ = 2.49 × 10^−4^), squamous cell lung carcinoma (OR = 1.098, 95% CI 1.021–1.181, *p* = 0.012, *P*_*fdr*_ = 0.012), and lung adenocarcinoma (OR = 1.098, 95% CI 1.024–1.178, *p* = 0.009, *P*_*fdr*_ = 0.012) are linked to an increased incidence of sepsis. Suggestive evidence was also found for small cell lung carcinoma (Wald ratio: OR = 1.156, 95% CI 1.047–1.277, *p* = 0.004) in relation to sepsis. The multivariate MR suggested that the partial impact of all LC subtypes on sepsis might be mediated through body mass index. Reverse analysis did not find a causal relationship (*p* > 0.05 and *P*_*fdr*_ > 0.05).

**Conclusion:**

The study suggests a causative link between LC and increased sepsis risk, underscoring the need for integrated sepsis management in LC patients.

## 1 Introduction

Sepsis is described as “a life‐threatening organ dysfunction caused by dysregulated host systemic inflammatory and immune response to infection”. (Singer et al., 2016).Sepsis remains a serious global health challenge (Tiru et al., 2015; Zhu et al., 2022). Sepsis impacted close to 50 million individuals globally and accounted for about 20% of global deaths before the COVID-19 pandemic (Rudd et al., 2020). Key factors contributing to the development of sepsis include the pathogen’s virulence, the site and type of infection, and host factors such as age, genetic predisposition, and comorbidities (Mao et al., 2013; Pan et al., 2017; Moon et al., 2023). Despite advancements in understanding its mechanisms, sepsis remains a significant challenge in healthcare due to its rapid progression and high mortality rate (Bauer et al., 2020). Early recognition and prompt management are crucial in improving patient outcomes.

Lung cancer (LC) is responsible for 2.2 million new cases each year, ranking as the world’s second most prevalent cancer. Furthermore, it is the primary cause of cancer-related mortality, resulting in approximately 1.79 million deaths annually (Sung et al., 2021; Thai et al., 2021; Huang et al., 2022). From a pathological classification perspective, LC can be roughly divided into two types: non-small cell lung cancer (NSCLC) and small cell lung cancer (SCLC). NSCLC primarily includes histological subtypes such as adenocarcinoma (LUAD) and squamous cell carcinoma (SqCLC). The relationship between LC and sepsis is complex and intricate (Pavon et al., 2013; Hensley et al., 2019; Mirouse et al., 2020; Moore et al., 2020; Shvetsov et al., 2021; Xia et al., 2022). Most research evidence supports the idea that LC poses a risk for sepsis (Pavon et al., 2013; Hensley et al., 2019; Moore et al., 2020; Xia et al., 2022). The immunosuppressive state induced by the tumor itself or by cancer treatments can make these patients more susceptible to infections (Pavon et al., 2013; Hensley et al., 2019; Xia et al., 2022). The results of a prospective study on the likelihood of sepsis following cancer showed a heightened risk of sepsis in cancer survivors, these cancers include lung cancer, breast cancer, prostate cancer, and other solid tumors, as well as hematological tumors (Moore et al., 2020). Additionally, studies have shown that the incidence and mortality rates of sepsis among cancer patients are higher, severe sepsis is associated with 8.5% of all cancer deaths, costing 3.4 billion dollars per year (Williams et al., 2004). However, some study results differ from this view; for example, a study suggests both complementary and antagonistic relationships between cancer and sepsis (Mirouse et al., 2020). Yurii B. Shvetsov and colleagues, in a multiethnic cohort study concerning the association between sepsis mortality and specific cancer sites and treatment types, found that lung cancer was associated with a significantly lower increase in sepsis mortality compared to non-sepsis mortality (Shvetsov et al., 2021).

Conversely, there is currently no consensus on whether sepsis increases the risk of cancer incidence. A multicenter observational study suggests that the incidence of sepsis does not alter the oncological and prognostic results in patients with epithelial ovarian cancer (Said et al., 2023). However, another study confirms that sepsis was significantly linked to a higher risk of nine types of cancer within 5 years after sepsis diagnosis, including LC (Liu Z. et al., 2019).

Given these inconsistent academic findings and the constraints of observational research in establishing cause-and-effect relationships, Mendelian randomization (MR) can provide insights into causality that observational studies lack. MR uses genetic variations as instrumental variables (IVs) derived from genomic-wide linkage analyses for causality inference. MR functions like a natural randomized controlled trial (RCT), offering more substantial evidence and less vulnerability to confounding factors than observational studies. MR is extensively used in cancer and disease research (Li and Wang, 2023; Xu et al., 2023). Hence, performing a bidirectional MR study could be critical in deciphering causal links between sepsis and LC, paving the way for better prevention and therapies.

## 2 Materials and methods

### 2.1 Study design

**FIGURE 1:**
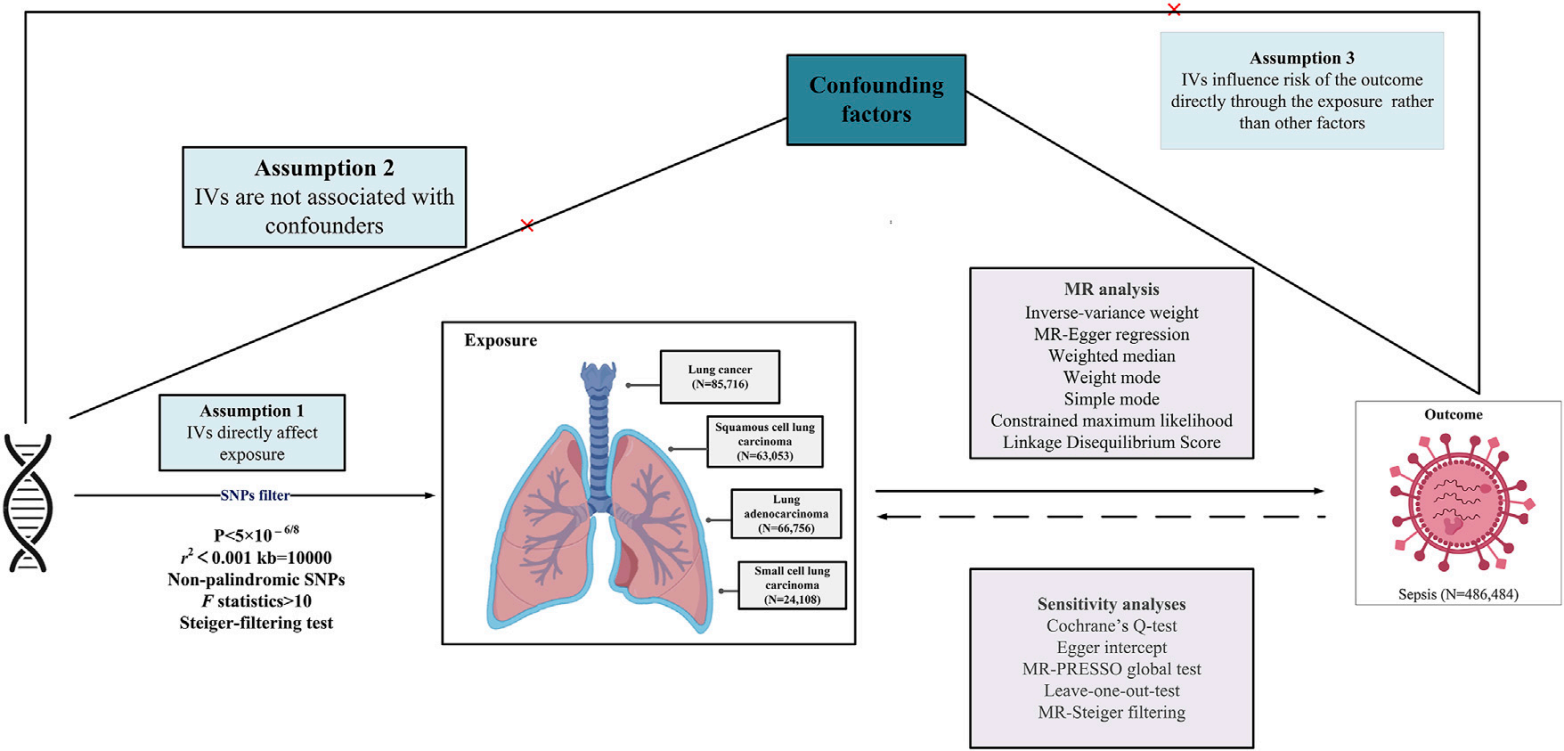
Overview of research design and analysis strategy. Overview of the research design. The MR framework is based on three fundamental MR assumptions. IVs, instrumental variables; MR, Mendelian randomization; MR-PRESSO, MR Pleiotropy Residual Sum and Outlier; SNP, single nucleotide polymorphism.

This study explores the causal relationship between LC and sepsis using summary-level data from the largest publicly accessible genome-wide association study (GWAS) currently available on these conditions. A suite of sophisticated analyses was conducted, incorporating bidirectional univariate MR, complementary multivariable MR (MVMR) analysis, and in-depth genetic correlation evaluations. IVs for exposure were established based on stringent criteria: (i) strong association of the genetic instrument with the exposure; (ii) independence of the instrument from confounding variables; (iii) the exclusive pathway of the genetic variants’ impact on the outcome is through the exposure (Lawlor et al., 2008). The methodological intricacies of the MR framework are presented in Figure 1, while the comprehensive summary data are systematically detailed in Table 1. This study is reported following the Strengthening the Reporting of Observational Studies in Epidemiology Using Mendelian Randomization guidelines (STROBE-MR) (Skrivankova et al., 2021).

**TABLE 1:** Detailed information of data sources.

Phenotypes	Ref	Ieu id	Consortium	Ancestry	Participants
**Exposure and outcome**					
LC	28604730	ebi-a-GCST004748	McKay JD	European	29,266 cases/56,450 controls
SqCLC	28604730	ebi-a-GCST004750	McKay JD	European	7,426 cases/55,627 controls
LUAD	28604730	ebi-a-GCST004744	McKay JD	European	11,273 cases/55,483 controls
SCLC	28604730	ebi-a-GCST004746	McKay JD	European	2,664 cases/21,444 controls
Sepsis	36402876	ieu-b-4980	United Kingdom Biobank	European	11,643 cases/474,841 controls
**Adjustment of the model**					
BMI	30124842	ieu-b-40	GIANT	European	681,275 individuals
EA	30038396	ieu-a-1239	SSGAC	European	1,131,881 individuals
T2DM	35551307	NA	DIAGRAM	European	80,154 cases/853,816 controls
ASI	30643251	ieu-b-4877	GSCAN	European	311,629 cases/321,173 controls
CPD	30643251	ieu-b-25	GSCAN	European	337,334 individuals
ADPW	30643251	ieu-b-73	GSCAN	European	335,394 individuals
COPD	36777996	NA	GBMI	European	58,559 cases/937,358 controls

ADPW, alcoholic drinks per week; ASI, age of smoking initiation; BMI, body mass index; COPD, chronic obstructive pulmonary disease; CPD, cigarettes per day; DIAGRAM, DIAbetes Genetics Replication And Meta-analysis; EA, education attainment; GBMI, Global Biobank Meta-analysis Initiative; GSCAN, GWAS, and Sequencing Consortium of Alcohol and Nicotine use; GIANT, genetic investigation of anthropometric traits; LC, lung cancer; LUAD, lung adenocarcinoma; Ref, reference (Pubmed id); SCLC, small cell lung carcinoma; SqCLC, squamous cell lung carcinoma; SSGAC, social science genetic association consortium; T2DM, type 2 diabetes mellitus.

### 2.2 Selection of genetic instrumental variables

The MR analysis operationalized rigorous selection parameters for Single nucleotide polymorphism (SNP) identification: (i) SNP, to serve as instrumental variables, showcased genome-wide significant associations with the exposure (*p* < 5 × 10^−8^). In the reverse analysis, due to the inability to obtain SNPs at the genome-wide significance level for the sepsis phenotype, we adjusted to a more relaxed threshold (*p* < 5 × 10^−6^) based on previous MR analysis experience to acquire a sufficient number of SNPs for the analysis (Liu et al., 2024; Xu et al., 2024; Yang et al., 2024). (ii) The selection of SNPs underwent rigorous scrutiny to exclude confounding variable associations and to confirm independence, thus preventing biases due to linkage disequilibrium (*r* ^2^ < 0.001, clumping distance = 10,000 kb). (iii) SNP validity as instrumental variables was gauged by F-statistics (F = *R* ^2^ ×(N− 2)/(1 − *R* ^2^)), where *R* ^2^ denotes the percentage of variance in the exposure explained by the SNPs, and N is the sample size of the GWAS from which the exposure is drawn). This criterion helped to eliminate weak instruments, with an F-statistic threshold of >10, ensuring robust instrument strength (Teumer, 2018). (iv) MR-Steiger filtering was applied to remove variants demonstrating stronger associations with outcomes than with exposures (Hemani et al., 2017). (v) In instances of SNP unavailability in the outcome dataset, the SNiPa web interface (http://snipa.helmholtz-muenchen.de/snipa3/) was employed, leveraging genotype data from the European cohort of the 1000 Genomes Project Phase 3, to locate a proxy SNP in strong linkage disequilibrium with the primary SNP (*r* ^2^ > 0.8). (vi) Consistency was essential, with the SNP’s effects on exposure and outcome required to be in the same allelic direction.

### 2.3 Source of lung cancer phenotype

This study utilizes the most extensive dataset to date, drawn from a meta-analysis by McKay JD et al., encompassing European ancestry GWAS for LC with 29,266 cases and 56,450 controls, SqCLC with 7,426 cases and 55,627 controls, LUAD with 11,273 cases and 55,483 controls, and SCLC with 2,664 cases and 21,444 controls (McKay et al., 2017). The research integrates novel data from the OncoArray genotyping platform with existing data from previous LC GWAS, conducting a large-scale association analysis on over 29,000 patients and 56,000 controls of European descent.

### 2.4 Source of sepsis phenotype

The latest and most exhaustive aggregate GWAS analysis about sepsis is derived from the UK Biobank (Sudlow et al., 2015). Methodological adjustments in this study included age, sex, ten principal genetic components, and genotyping batch effects. It comprised 11,643 sepsis cases juxtaposed against 474,841 controls, all of European descent. Case identification hinged on the presence of ICD-10 codes A02, A39, A40, and A41.

### 2.5 MVMR models

Lung cancer and sepsis share common risk factors, including smoking, chronic obstructive pulmonary disease (COPD), body mass index (BMI), chronic conditions like type 2 diabetes mellitus (T2DM), and other factors such as education level (Biesalski et al., 1998; Pallis and Syrigos, 2013; Henriksen et al., 2015; Bohl et al., 2016; Malhotra et al., 2016; Locham et al., 2021; Bladon et al., 2024). Considering potential confounding factors, seven major confounding factors were selected. BMI data was sourced from the Genetic Investigation of Anthropometric Traits (GIANT) consortium (Yengo et al., 2018). Educational attainment (EA) data came from the Social Science Genetic Association Consortium (SSGAC) (Lee et al., 2018). T2DM data was acquired from the DIAbetes Genetics Replication And Meta-analysis (DIAGRAM) consortium (Mahajan et al., 2022). Data on cigarettes per day (CPD), age of smoking initiation (ASI), and alcoholic drinks per week (ADPW) were derived from the GWAS and Sequencing Consortium of Alcohol and Nicotine Use (GSCAN) (Liu M. et al., 2019). COPD data came from the Global Biobank Meta-analysis Initiative (GBMI) (Zhou et al., 2022).

### 2.6 Statistical analyses

#### 2.6.1 MR analysis

The univariate MR framework evaluated individual IVs using the Wald ratio, which calculates the causal effect by dividing SNP-outcome association (β_Y) by the SNP-exposure association (β_X). This method provides a causal estimate for each genetic variant, assuming IVs significantly influence the exposure, are independent of confounders, and affect the outcome solely through the exposure (Burgess et al., 2013). Concurrently, to elucidate the causal associations involving multiple IVs (two or more), use the multiplicative random-effects inverse-variance-weighted (IVW) method (Burgess et al., 2013). It is critical to note that when the heterogeneity index I^2^ is below 50%, outcomes derived from the fixed-effects model are considered robust. This statistical strategy was additionally refined by incorporating the MR-Egger and weighted median methodologies. The IVW technique’s weighting schema is coherent with the Wald ratio estimates for each SNP, inversely correlating with its variance (Hemani et al., 2018). By integrating the full complement of genetic variants, the IVW approach ensures systematic and reliable results. Contrariwise, the weighted median method gains prominence when more than half of the genetic variants are presumed invalid; concurrently, the MR-Egger method operates under the premise that all such variants are invalid (Bowden et al., 2016). Furthermore, the constrained maximum likelihood (CML) process was employed, allowing for collective analysis over an expansive array of genetic variants while adjusting for possible confounders and intrinsic genetic heterogeneity. Particularly when addressing a comprehensive array of genetic variants and confounders, the CML method is indispensable for obtaining accurate and robust results (Zhang et al., 2008).

Additional MVMR analyses were carried out to delineate the direct causal pathways from exposure to outcome (Burgess and Thompson, 2015). These analyses were to define the direct causal connections precisely, thereby differentiating them from the univariate MR model. Contrary to UVMR, which concentrates on a singular exposure, MVMR considers genetic variations linked to multiple exposures. The initial stage involved generating Mendelian Randomization effect estimates for the exposure-to-outcome relationships using the IVW method. Subsequently, an MVMR assessment was carried out to assess the impact of six mediators on the outcome, considering the specific attributes of the exposure.

This study conducted its analysis using the “MendelianRandomization,” “TwoSampleMR,” “MR-PRESSO,” and “MRMR” packages in R version 4.3.0 software.

#### 2.6.2 LDSC regression analysis

The linkage disequilibrium score (LDSC) regression, designed for analyzing GWAS summary data, constitutes an effective instrument for discerning genetic correlations among complex diseases or traits. This method facilitates the separation of authentic polygenic influences from confounding factors, which include subtle family structures and population stratification (Bulik-Sullivan et al., 2015). A significant genetic correlation, characterized by statistical solidity and substantial effect size, implies that the correlation between phenotypes is not merely attributable to environmental influences. The LDSC, available at (https://github.com/bulik/ldsc), provides a direct path for investigating the genetic foundations linking exposure and outcome traits.

#### 2.6.3 Sensitivity analysis

Diversity among chosen genetic variants was measured using Cochran’s Q test, with a *p*-value less than 0.05, denoting notable differences within the SNPs under study (Kulinskaya et al., 2020). MR-Egger regression was utilized to investigate directional pleiotropy within the MR context (Burgess and Thompson, 2017). An MR-Egger intercept with a *p*-value below 0.05 indicates notable directional pleiotropy despite the acknowledged limitations of this method (Wu et al., 2020). The MR Pleiotropy Residual Sum and Outlier (MR-PRESSO) approach was used to pinpoint outliers and evaluate horizontal pleiotropy, with a global *p*-value below 0.05 confirming its presence (Verbanck et al., 2018). Outliers were rigorously removed to refine analytical accuracy. This was followed by a leave-one-out sensitivity analysis to appraise the effect of single SNPs on the collective outcomes (Cheng et al., 2017). The false discovery rate (FDR) method was employed to correct multiple comparisons rigorously. Post-correction, *p*-values less than 0.05 denoted significant causal associations. Conversely, outcomes with raw *p*-values under 0.05 that did not maintain this significance after FDR adjustment were classified as suggestive rather than definitive.

For the calculation of *R* ^2^ values, equation 2×MAF×(1-MAF)×beta^2^ was used, where MAF represents the minor allele frequency for each SNP. These calculated *R* ^2^ values were then gathered to establish the combined parameter for power calculation (Guan et al., 2014). The mRnd platform (Brion et al., 2013) (https://shiny.cnsgenomics.com/mRnd/) provided the means for statistical power assessment.

## 3 Results

### 3.1 Genetic instrument selection and genetic correlation between phenotypes

**FIGURE 2:**
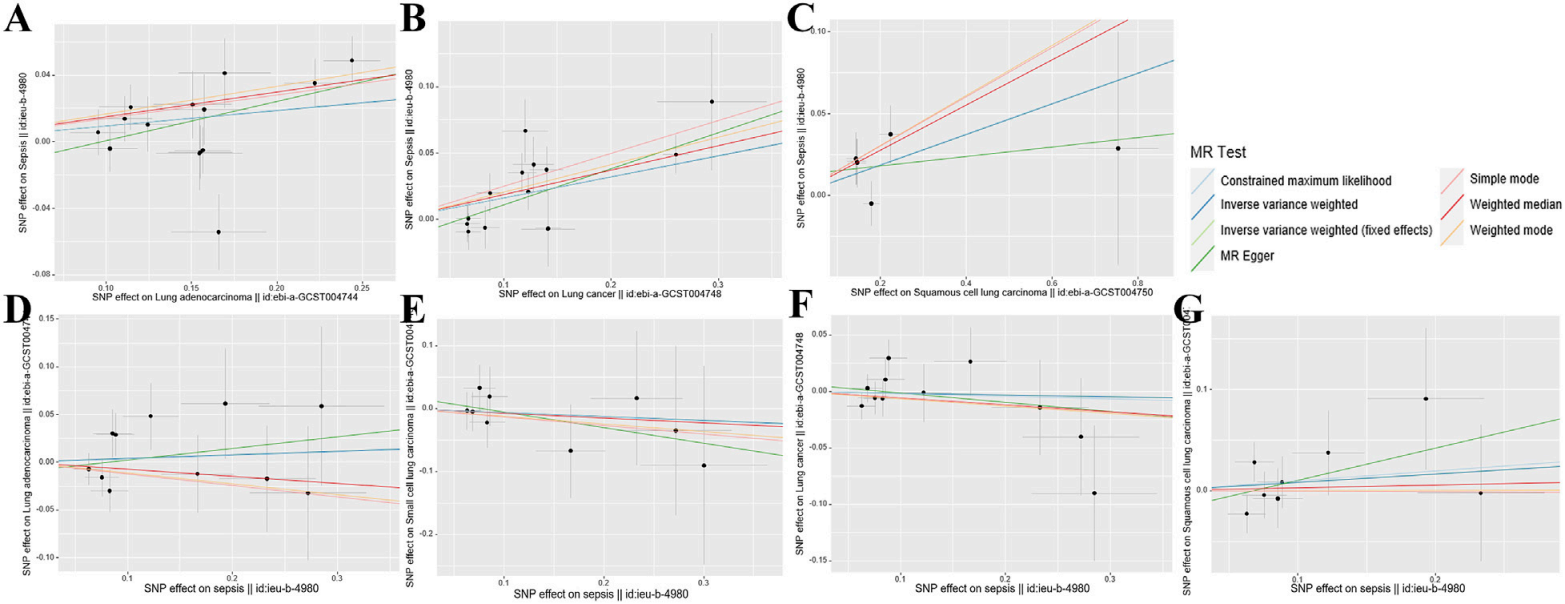
Scatterplot summary of all MR analyses. The vertical and horizontal lines denote the 95% confidence intervals for the effect size, while the slopes of the fitted lines indicate the estimated Mendelian randomization effect per method. **(A)** Lung adenocarcinoma on sepsis **(B)** Lung cancer on sepsis **(C)** Squamous cell lung carcinoma on sepsis **(D)** Sepsis on lung adenocarcinoma **(E)** Sepsis on small cell lung carcinoma **(F)** Sepsis on lung cancer **(G)** Sepsis on squamous cell lung carcinoma. MR, Mendelian randomization; SNP, single nucleotide polymorphism.

The research findings suggest that the F-statistics for all instrumental variables surpassed 500, suggesting a significant decrease in bias due to weak instrument variation. The quantity of SNPs selected as instrumental variables ranged from one to 14, with the explained variance in genetic variation ranging from 1.75% to 12.36% (Supplementary Table S1). The scatter plot (Figure 2) provides an intuitive representation of the direction of causal associations, while the forest plot (Supplementary Figure S1) displays the effects contributed by all IVs. Detailed SNP information can be found in Supplementary Tables S2–S8.

The LDSC study uncovered a substantial genetic link between LC and sepsis (r_g_ = 0.325, *p* = 0.014); however, no genetic correlation was detected between the subtypes of LC: SqCLC (r_g_ = 0.095, *p* = 0.605), LUAD (r_g_ = 0.295, *p* = 0.052), and SCLC (r_g_ = 0.151, *p* = 0.305), and sepsis. The SNP-based liability-scale heritability (h^2^) ranged from 0.24% to 10.13% (Supplementary Table S3).

### 3.2 Association of genetically predicted lung cancer with sepsis

**FIGURE 3:**
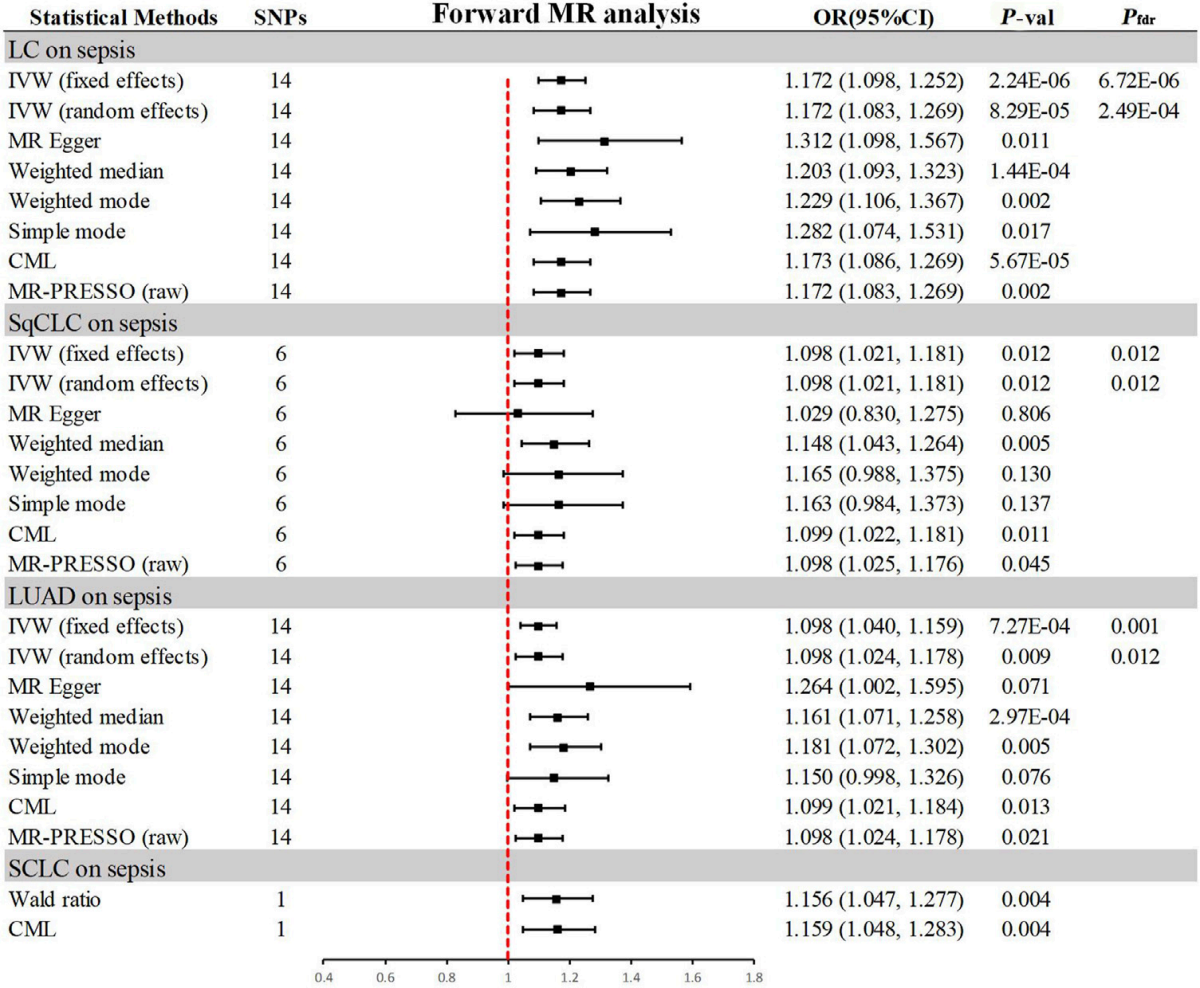
Summary of results from MR analysis of genetically predicted lung cancer phenotypes for sepsis. CML, constrained maximum likelihood; FDR, false discovery rate; IVW, inverse-variance-weighted; LC, lung cancer; LUAD, lung adenocarcinoma; MR, Mendelian randomization; MR-PRESSO, MR Pleiotropy Residual Sum and Outlier; OR, odd ratio; *P*-val, *p*-value; SCLC, small cell lung carcinoma; SqCLC, squamous cell lung carcinoma; SNP, single nucleotide polymorphism.

In the progressive MR analysis (Figure 3), a significant causal relationship was identified between all subtypes of LC and sepsis. Expressly, after FDR correction for multiple comparisons, the primary analysis method, IVW, indicated that each standard deviation (SD) increase in genetically predicted LC was linked to a 17.2% increase in the risk of sepsis, with both random (OR = 1.172, 95% CI 1.083–1.269, *p* = 8.29 × 10^−5^, *P*_*fdr*_ = 2.49 × 10^−4^) and fixed (OR = 1.172, 95% CI 1.098–1.252, *p* = 2.24 × 10^−6^, *P*_*fdr*_ = 6.72 × 10^−6^, I^2^ = 30%) effects models showing concordance. Supplementary methods provided consistent evidence of this causal relationship. With an OR of 1.172, we achieved 100% statistical power to detect the association between LC and sepsis. Among LC subtypes, for each SD increase in genetically predicted SqCLC, the risk of sepsis increased by 9.8%, with IVW random (OR = 1.098, 95% CI 1.021–1.181, *p* = 0.012, *P*_*fdr*_ = 0.012) and fixed (OR = 1.098, 95% CI 1.021–1.181, *p* = 0.012, *P*_*fdr*_ = 0.012, I^2^ = 0%) effects models in agreement. Supplementary methods, including the Weighted median (OR = 1.148, 95% CI 1.043–1.264, *p* = 0.005) and CML (OR = 1.099, 95% CI 1.022–1.181, *p* = 0.011), also yielded consistent causal evidence. At an OR of 1.098, we possessed 78% statistical strength to identify the relationship between SqCLC and sepsis. Similarly, for each SD increase in genetically predicted LUAD, there was a 9.8% increase in sepsis risk, with concordance across random (OR = 1.098, 95% CI 1.024–1.178, *p* = 0.009, *P*_*fdr*_ = 0.012) and fixed (OR = 1.098, 95% CI 1.040–1.159, *p* = 7.27 × 10^−4^, *P*_*fdr*_ = 0.001, I^2^ = 39%) effects models in IVW and consistency in supplementary methods excluding the simple mode (OR = 1.150, 95% CI 0.998–1.326, *p* = 0.076). With an OR of 1.098, we had a 96% statistical strength to determine the relationship between LUAD and sepsis. Finally, in the case of SCLC and sepsis, due to the limited IVs available for analysis, only Wald ratio (OR = 1.156, 95% CI 1.047–1.277, *p* = 0.004) and CML (OR = 1.159, 95% CI 1.048–1.283, *p* = 0.004) were used, which also showed a 100% statistical strength to identify the association. However, due to the inability to perform multidimensional validation, the evidence indicated a suggestive risk.

In the sensitivity analysis (Table 2), all IVs passed the MR-Steiger filtering, and Cochran’s Q statistic indicated no significant heterogeneity (*p* > 0.05). Likewise, MR-Egger and MR-PRESSO tests revealed no pleiotropy (*p* > 0.05). Leave-one-out analysis confirmed that the causal inference was not driven by any single SNP (Supplementary Figure S2), and the funnel plot exhibited a symmetrical distribution (Supplementary Figure S3).

**TABLE 2:** Summary of sensitivity results.

Exposure	Outcome	MR-egger intercept			MR-PRESSO global test		Cochrane’s Q				Steiger_test	
		Intercept	SE	P-val	RSSobs	P-val	Q	Q_df	Q_P-val	I2 (%)	Direction	P-val
Forward MR analysis												
LC	Sepsis	−0.016	0.012	0.194	21.537	0.179	18.775	13	0.130	30	TRUE	1.06E-185
SqCLC	Sepsis	0.012	0.020	0.564	7.193	0.470	4.493	5	0.481	0	TRUE	9.25E-64
LUAD	Sepsis	−0.023	0.018	0.237	25.984	0.067	21.527	13	0.063	39	TRUE	4.08E-155
SCLC	Sepsis	-	-	-	-	-	-	-	-	0	TRUE	1.15E-17
Reverse MR analysis												
Sepsis	LC	0.007	0.013	0.615	10.745	0.544	8.859	10	0.545	0	TRUE	8.10E-04
Sepsis	SqCLC	−0.022	0.027	0.458	7.313	0.599	5.591	7	0.588	0	TRUE	0.028
Sepsis	LUAD	−0.010	0.019	0.629	11.394	0.482	9.481	10	0.487	0	TRUE	0.011
Sepsis	SCLC	0.020	0.034	0.581	3.050	0.966	2.404	8	0.966	0	TRUE	0.124

df, degree of freedom; LC, lung cancer; LUAD, lung adenocarcinoma; MR, mendelian randomization; MR-PRESSO, MR, pleiotropy residual sum and outlier; P-val, *p*-value; RSSobs, residual sum of squares observation; SCLC, small cell lung carcinoma; SE, standard error; SqCLC, squamous cell lung carcinoma.

### 3.3 Association of genetically predicted sepsis with lung cancer

**FIGURE 4:**
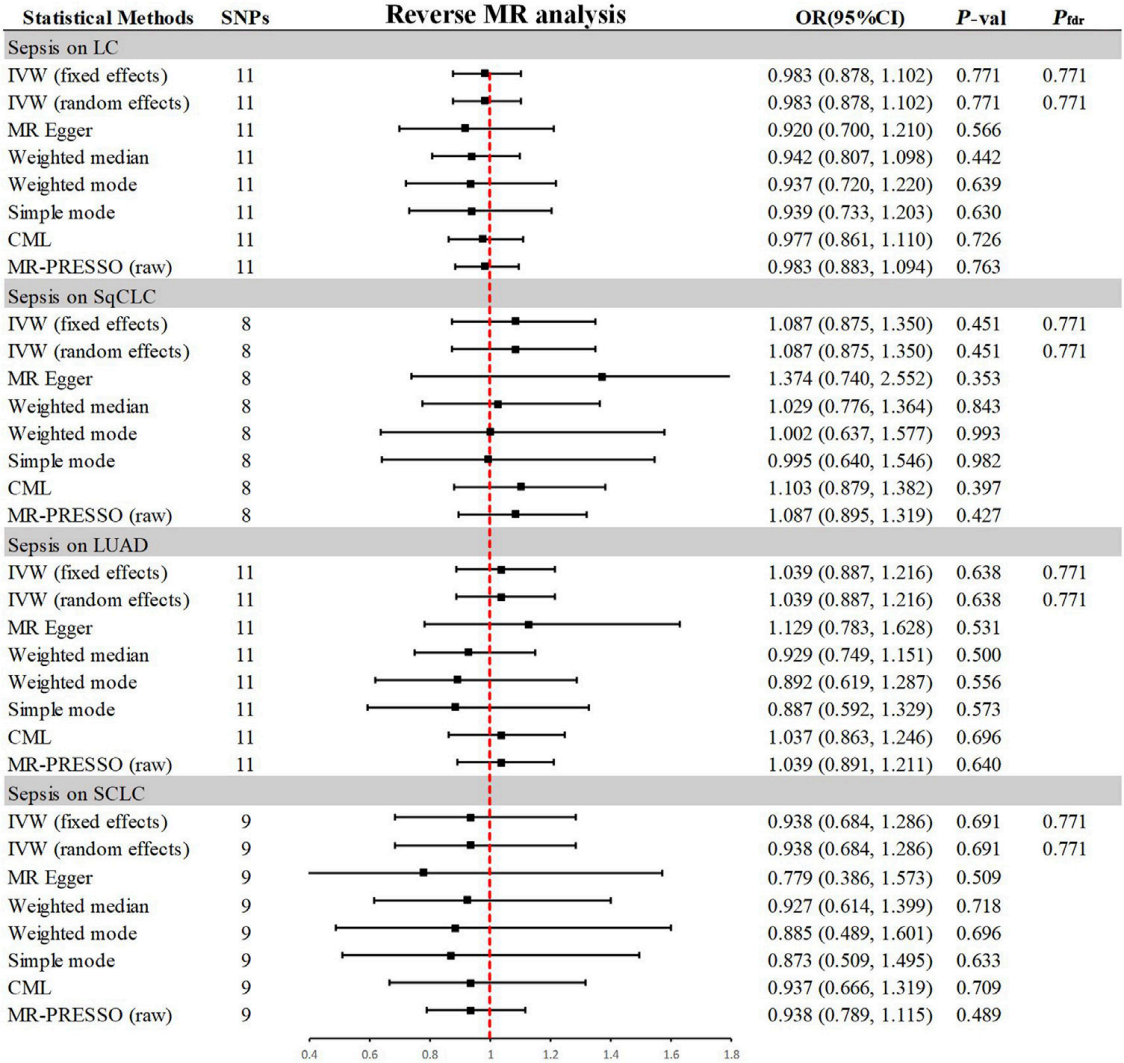
Summary of results from MR analysis of genetically predicted sepsis for lung cancer phenotypes. CML, constrained maximum likelihood; FDR, false discovery rate; IVW, inverse-variance-weighted; LC, lung cancer; LUAD, lung adenocarcinoma; MR, Mendelian randomization; MR-PRESSO, MR Pleiotropy Residual Sum and Outlier; OR, odds ratio; *P*-val, *p*-value; SCLC, small cell lung carcinoma; SqCLC, squamous cell lung carcinoma; SNP, single nucleotide polymorphism.

In the reverse MR analysis (Figure 4), the primary method, IVW, indicated no reverse causal effects between sepsis and LC (OR = 0.983, 95% CI 0.878–1.102, *p* = 0.771, *P*_*fdr*_ = 0.771), SqCLC (OR = 1.087, 95% CI 0.875–1.350, *p* = 0.451, *P*_*fdr*_ = 0.771), LUAD (OR = 1.039, 95% CI 0.887–1.216, *p* = 0.638, *P*_*fdr*_ = 0.771), or SCLC (OR = 0.938, 95% CI 0.684–1.286, *p* = 0.691, *P*_*fdr*_ = 0.771). Additional supplementary methods provided consistent evidence supporting these associations (*p* > 0.05). Comprehensive sensitivity analyses confirmed the robustness of the findings (*p* > 0.05), as detailed in Table 2. Corresponding figures are provided in Supplementary Figures S2–S3.

### 3.4 MVMR analysis

In the univariate MR analysis, evidence supported a causal relationship between LC and its subtypes and the risk of sepsis, reaching statistical significance (*p* < 0.05 and *P*_*fdr*_ < 0.05). Since only one IV related to SCLC was obtained, we excluded the SCLC phenotype from further analysis to avoid biased results in MVMR adjustments. In the MVMR analysis (Table 3), the causal link between LC and sepsis was no longer notable when adjusting for BMI (OR = 1.023, 95% CI 0.944–1.108, *p* = 0.581). Similarly, SqCLC adjusted for BMI (OR = 1.031, 95% CI 0.975–1.091, *p* = 0.279), EA (OR = 1.052, 95% CI 0.990–1.118, *p* = 0.099), T2DM (OR = 1.070, 95% CI 0.992–1.154, *p* = 0.081), and LUAD adjusted for BMI (OR = 0.989, 95% CI 0.923–1.060, *p* = 0.752) and CPD (OR = 1.060, 95% CI 0.989–1.136, *p* = 0.101) also showed no significant causal relationship with sepsis. This suggests that these confounding factors may partially mediate the causal relationship between LC, subtypes, and sepsis.

**TABLE 3:** Summary of analytical results for MVMR.

Exposure	MVMR models	SNP	*P*-val	OR (95%CI)
LC on sepsis	BMI	449	0.581	1.023 (0.944, 1.108)
	EA	265	0.001	1.123 (1.050, 1.200)
	T2DM	161	0.003	1.069 (1.024, 1.116)
	ASI	82	5.18E-07	1.193 (1.114, 1.278)
	CPD	29	0.003	1.145 (1.047, 1.252)
	ADPW	44	1.50E-06	1.183 (1.105, 1.267)
	COPD	25	0.012	1.128 (1.027, 1.239)
SqCLC on sepsis	BMI	458	0.279	1.031 (0.975, 1.091)
	EA	258	0.099	1.052 (0.990, 1.118)
	T2DM	151	0.081	1.070 (0.992, 1.154)
	ASI	84	0.025	1.079 (1.010, 1.153)
	CPD	26	0.023	1.086 (1.011, 1.167)
	ADPW	36	0.007	1.110 (1.029, 1.196)
	COPD	22	0.030	1.087 (1.008, 1.172)
LUAD on sepsis	BMI	449	0.752	0.989 (0.923, 1.060)
	EA	269	0.001	1.103 (1.041, 1.170)
	T2DM	158	5.50E-05	1.143 (1.071, 1.220)
	ASI	85	0.001	1.104 (1.042, 1.170)
	CPD	28	0.101	1.060 (0.989, 1.136)
	ADPW	42	0.002	1.103 (1.035, 1.175)
	COPD	21	0.048	1.206 (1.002, 1.451)

ADPW, alcoholic drinks per week; ASI, age of smoking initiation; BMI, body mass index; CI, confidence interval; COPD, chronic obstructive pulmonary disease; CPD, cigarettes per day; EA: education attainment; LC, lung cancer; LUAD, lung adenocarcinoma; MVMR, multivariable mendelian randomization; OR, odds ratio; *P*-val, *p*-value; SNP, single nucleotide polymorphism; SqCLC, squamous cell lung carcinoma; T2DM, type 2 diabetes mellitus.

## 4 Discussion

This research undertook a comprehensive MR analysis to explore the link between genetic predisposition to sepsis and LC. The results of the MR support earlier epidemiological research (Pavon et al., 2013; Rhee et al., 2017; Xia et al., 2022), confirming a causal link between sepsis and LC. Furthermore, no reverse causal link was found between LC and sepsis. Additional MVMR analysis suggested that factors such as body mass index, level of education, type 2 diabetes, and information on daily cigarette consumption might play a role in mediating part of this causative link.

Prior studies have indicated a link between sepsis and LC, with results showing a correlation between LC and an increased likelihood of sepsis (Pavon et al., 2013; Hensley et al., 2019; Xia et al., 2022). The immunosuppressive state induced by the tumor itself or by cancer treatments can make these patients more susceptible to infections (Pavon et al., 2013). In a group of more than one million hospital admissions for sepsis in the U.S., over 20% had a connection to cancer (Hensley et al., 2019). The results of a prospective study on the risk of sepsis after cancer showed an increased risk of sepsis in cancer survivors (Moore et al., 2020). Nonetheless, previous research has yielded inconsistent results concerning the relationship between general sepsis and LC. Notably, one study demonstrated that the relationship between cancer and sepsis is complementary and antagonistic (Mirouse et al., 2020). Our results are similar to the results of previous mainstream studies. Our MR results support the idea that LC contributes to sepsis.

Alternatively, there is currently no consensus on whether sepsis increases the risk of cancer incidence. A multicenter observational study suggests that the occurrence of sepsis does not affect the oncological and survival outcomes in patients with epithelial ovarian cancer (Said et al., 2023). However, another study confirms that sepsis was notably linked with a heightened risk of nine different cancer types in 5 years after a sepsis diagnosis, including LC (20). In our study, sepsis was not found to act as a genetic predisposing element for LC.

Based on previous disparities in research and incorporating the findings of this study, we posit that, given cancer and sepsis are not singularly unique diseases, it is evident that the risk associated with breast cancer differs from that of pancreatic cancer. Biological distinctions also exist between solid malignancies and malignancies of the hematopoietic system. Consequently, categorizing all cancer or sepsis patients uniformly is erroneous, and treatments conducted in this context are likely to yield suboptimal outcomes. Sepsis, akin to cancer, exhibits intricate diversity. Gaining a deeper understanding of the distinctive physiological states induced by sepsis and cancer is complex and crucial.

Observational studies often face limitations due to unobserved confounding factors and reverse causality, focusing more on correlation than causation. While data indicates a connection, the causal link between sepsis and LC has not yet been conclusively proven. We used MR analysis to investigate the genetic underpinnings of the causative link between sepsis and LC to counteract biases and confounders. Our study indicates that we must pay attention to monitoring patients with LC infection and inflammation factors and prevention and intervention in promptly treating sepsis.

Further MVMR analyses underscored the significance of BMI, educational attainment, T2DM, and data on cigarettes per day. Firstly, high BMI, especially obesity, may increase the risk of infection because obesity may affect the function of the immune system and may be associated with chronic inflammation. An MR study also showed that obesity was linked to a heightened likelihood of developing sepsis (Hu et al., 2023). Secondly, educational attainment may indirectly affect an individual’s risk of sepsis by influencing their lifestyle, health behaviors, access to medical resources, and the environment in which they live and work. For example, previous observational research has shown that lower educational attainment (EA) levels are connected to a heightened likelihood of COVID-19 (Jian et al., 2021). Additionally, individuals with diabetes have a higher propensity to develop wounds and ulcers that do not heal and can become infected, resulting in sepsis. In addition, diabetes alters the immune system, leading to an increased risk of sepsis (Schuetz et al., 2011). Lastly, smoking may elevate infection risk by increasing proinflammatory cytokines, damaging endothelial cells, and correlates with poor health habits (Alroumi et al., 2018; Zhang et al., 2022). A causative link between smoking and infectious disease risk was also shown in an MR study (Zhu et al., 2023). Therefore, this implies a comprehensive strategy for managing sepsis, considering these factors combined.

Our research has several advantages. This MR study represents the inaugural exploration of the causative link between sepsis and LC at the genetic level. All the SNPs selected as instrumental variables (IVs) originated from the European demographic, thus diminishing the probability of population stratification bias and bolstering the credibility of the bidirectional MR hypothesis. Our robust tools in this research (such as an F statistic significantly exceeding 10) should mitigate potential bias from sample overlap. Nonetheless, our investigation has its limitations. Several initial exposures were sourced from the UKB cohort, and the absence of additional GWAS hindered the execution of a confirmatory control analysis. Additionally, the exclusive access to summary-level GWAS data impeded the conduct of more detailed subgroup analyses.

In conclusion, our study used a comprehensive approach to investigate the association between lung cancer and sepsis, providing novel insights. Our results suggest that LC is a significant risk factor for sepsis, however, sepsis was not found to act as a genetic predisposing factor for LC. Further in-depth research is warranted to unravel the additional intricacies of this relationship. These efforts underscore the need for integrated sepsis management in LC patients.

## 5 Conclusion

To summarize, our study establishes a causal relationship between LC and increased risk of sepsis, with no evidence for a reverse association. Comprehensive prevention and treatment of sepsis should be carried out in LC patients, especially those with high BMI, low educational attainment, T2DM, and smoking.

## Data Availability

The original contributions presented in the study are included in the article/Supplementary Material, further inquiries can be directed to the corresponding author.

## Abbreviations

ADPW, alcoholic drinks per week; ASI, age of smoking initiation; BMI, body mass index; CI, confidence interval; CML, constrained maximum likelihood; COPD, chronic obstructive pulmonary disease; CPD, cigarettes per day; df, degree of freedom; DIAGRAM, DIAbetes Genetics Replication And Meta-analysis; EA, education attainment; GBMI, Global Biobank Meta-analysis Initiative; GIANT, Genetic Investigation of Anthropometric Traits; GSCAN, GWAS and Sequencing Consortium of Alcohol and Nicotine use; IVs, instrumental variables; LC, lung cancer; LUAD, lung adenocarcinoma; MR, Mendelian Randomization; MR-PRESSO, MR Pleiotropy Residual Sum and Outlier; MVMR, Multivariable Mendelian randomization; OR, odds ratio; P-val, *p*-value; RSSobs, residual sum of squares observation; SCLC, small cell lung carcinoma; SNP, single nucleotide polymorphism; SqCLC, squamous cell lung carcinoma; SSGAC, Social Science Genetic Association Consortium; T2DM, type 2 diabetes mellitus.
